# Public awareness, prevalence, and regulations for the sale of electronic cigarettes in Arab countries: A narrative review

**DOI:** 10.18332/tid/168435

**Published:** 2023-10-27

**Authors:** Feras Jirjees, Yahya H. Dallal Bashi, Zelal Kharaba, Keivan Ahmadi, Muna Barakat, Hala AlObaidi

**Affiliations:** 1 College of Pharmacy, University of Sharjah, Sharjah, United Arab Emirates; 2 School of Pharmacy, Queen’s University Belfast, Belfast, United Kingdom; 3 College of Pharmacy, Al Ain University, Abu Dhabi, United Arab Emirates; 4 Faculty of Medical Sciences, Newcastle University, Newcastle, United Kingdom; 5 NIHR Applied Research Collaboration Northwest London, Department of Primary Care and Public Health, School of Public Health, Faculty of Medicine, Imperial College London, London, United Kingdom; 6 Department of Clinical Pharmacy and Therapeutics, School of Pharmacy, Applied Science Private University, Amman, Jordan

**Keywords:** e-cigarettes, smoking, vaping, awareness, Arab, regulation

## Abstract

The majority of the Arab countries have high records of daily tobacco smoking. Electronic cigarettes (e-cigarettes) use has exponentially increased in the past decade in Arab countries. Consumers’ willingness to quit smoking traditional cigarettes and their perception of e-cigarettes as a safer alternative to conventional cigarettes have increased the demand for these devices. This narrative review aimed to gather and discuss the available literature on the awareness, prevalence, and sales regulations of e-cigarettes in Arab countries. A search was conducted on electronic databases such as PubMed, Medline, Scopus, and Google Scholar with no time limits until the end of 2021. Some of the recent studies (2019–2021) considered in this review have reported that more than 25% of participants were e-cigarettes users. The prevalence of e-cigarettes has increased drastically with raised awareness of e-cigarettes among the population in these Arab countries. Most of the users of e-cigarettes are young males. In addition, an increase in e-cigarettes use has been reported in females. The policies which regulate trade and sale of e-cigarettes and related products were issued in only three Arab countries. In contrast, regulations for the trade and sale of traditional cigarette products are also applied in some countries to e-cigarette products, banning the sale of e-cigarettes to minors and/or amend smoke-free laws to restrict public use of e-cigarettes. There is a need for a concerted effort to assess the prevalence and significant rise of e-cigarettes consumption in Arab societies to help implement and improve harm reduction policies.

## INTRODUCTION

The harmful effects of smoking tobacco, in most methods, are well-established and this can affect nearly every organ in the body, as well as the overall health status^1^. As a result, governments and health systems have issued regulations to control smoking to reduce smoking prevalence. Although there is no strong evidence that one method of smoking is less harmful than another, it has been argued that some smoking methods contain lower levels of chemicals such as nicotine and then less risk to health than others^2^.

Electronic cigarettes (e-cigarettes) entered the market about two decades ago, and their use has risen rapidly with a multi-fold increase in users worldwide^2-6^. E-cigarettes heat a liquid (that may contain nicotine) to create inhaled vaporised aerosols. These e-cigarette products have been marketed as less harmful than other smoking methods^7^. However, recent literature has shown controversial findings and insufficient scientific evidence about the safety of e-cigarettes and the associations between perceived harm and potential adverse effects^8-10^. In addition, several studies have identified findings of the long-term benefit-to-harm ratio of e-cigarettes to consume nicotine over traditional tobacco smoking. That is because they may serve as a gateway to smoking in the future, given their low perceived risk or their use may prevent regular smokers from quitting smoking by maintaining nicotine addiction^11,12^. The proposed harm reduction of e-cigarettes as a smoking cessation strategy has encouraged some smokers to take up e-cigarettes to quit traditional cigarettes successfully; however, there is no sufficient evidence to support this claim fully. Governments and health systems have responded in various ways to such claims, as some countries accepted and regulated e-cigarettes sales, and other countries banned the sale of e-cigarettes. In 2017, out of 68 countries, 22 countries were controlling e-cigarettes by using the existing regulations on other smoking products; 25 countries put new policies in place to regulate e-cigarette sales, seven countries made some changes to the current legislation for smoking products to accommodate e-cigarettes regulations, and 14 countries were using a combination of new rules and/or the existing regulations and/or existing modified regulations^13^.

The vaping behaviour and its prevalence are dependent upon numerous factors such as the government and health sector policies and regulations on the sales and consumption of e-cigarettes, public awareness and attitudes towards using e-cigarettes as a harm reduction strategy, and the availability of e-cigarette devices and e-liquids in the market^10,14,15^. Perceptions of the population regarding e-cigarettes impact their desire to use e-cigarettes. The most common perceptions that make e-cigarettes more acceptable in the population include quitting smoking, as they are marketed to be less harmful than traditional tobacco smoking. Moreover, e-cigarettes are marketed with different e-liquid flavours and varied nicotine contents, which might be more desirable to youth smokers. However, there are not enough data on long-term abstinence from smoking due to e-cigarettes^16^.

In the Middle East and North Africa (MENA) region, the largest relative increase (104.1%) in the number of smokers in the past 30 years was recorded. The prevalence of tobacco smoking is high in several countries, with averages of 32.4% and 5.63% among males and females aged ≥15 years, respectively. Ten Arab countries (out of 18 countries in the study) have recorded higher tobacco smoking rates than previously, with the highest percentage being recorded in Jordan among males (53%) and in Lebanon among females (26%)^17^. Although e-cigarettes use in the MENA region was reported a decade ago, there are insufficient data on the prevalence of e-cigarette use in many Arab countries.

This narrative review aimed to study and analyze the published literature that has reported the prevalence, awareness, regulations, marketing/sales, and consumption patterns of e-cigarettes among the Arab population.

Within this narrative review, we searched four databases PubMed, Medline, Scopus, and Google Scholar, until the end of December 2021, using the following keywords: ‘electronic cigarette’, ‘e-cigarette’, ‘vaping’, ‘vape’, ‘e-cigs’, ‘electronic nicotine delivery systems’, ENDS, ‘vaporizer cigarettes’, ‘vape pens’, their Arab words, and names of 21 Arab countries. Inclusion criteria were studies conducted in Arab countries related to the prevalence of e-cigarette use, awareness among users of e-cigarettes, and/or e-cigarette sales, and consumption regulations.

Two reviewers (FJ and HA) screened titles and abstracts of published articles against the described eligibility criteria. Any discrepancies were resolved via discussion or the inclusion of a third reviewer (KA). FJ, HA and MB appraised the methodological quality of the studies independently, using the McGill Mixed-Methods Appraisal Tool (MMA)^18^. Any discrepancies were resolved via discussion between FJ and HA. FJ, MB and YDB extracted the regulation policies related to trade and sales of e-cigarettes from the official websites such as Ministries of Health, customs authorities, and Ministries of Finances in the Arab countries.

**Figure 1:**
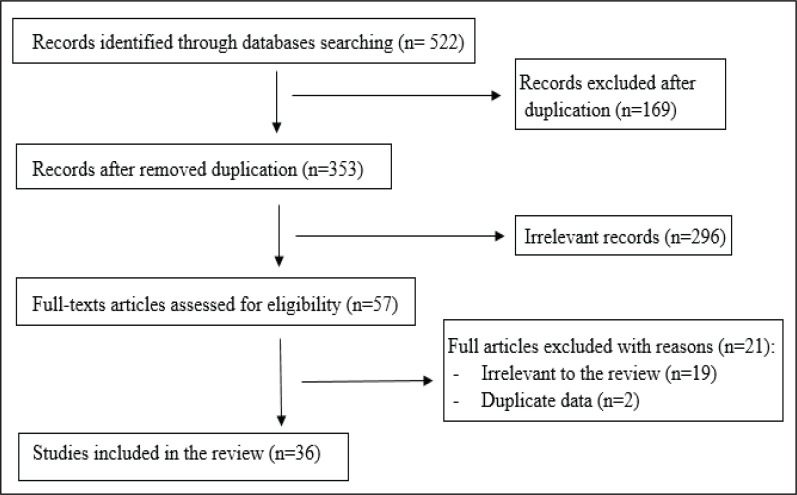
A flow chart of selection of studies

The search initially resulted in 353 e-cigarette-related publications following the removal of duplicates. Then, 296 articles were excluded due to irrelevance to this review’s topic and/or aim. Finally, 36 studies were included as shown in Figure 1.

All the included studies (n=36) were cross-sectional, survey-based studies. The sample size of these studies ranged between 193 and 8389 participants. These studies related to e-cigarettes behaviour and awareness were carried out in seven Arab countries, including Saudi Arabia, Jordan, Egypt, United Arab Emirates (UAE), Lebanon, Tunisia, and Qatar^19-54^. These studies were published between 2014 and 2021. Different populations were included in these selected studies; the general population (n=19), students (n=16), and pregnant women (n=1) (Table 1). Furthermore, 16 studies were used to extract data on the prevalence of e-cigarettes among people in Arab countries in addition to the included studies.

**Table 1:** Summary of the studies related to knowledge and/or awareness among participants about e-cigarette use in the Arab societies

*Study Year*	*Country*	*Number of participants*	*Age (years)*	*Gender (% male)*	*Education level of the participants*	*Main outcomes related to awareness/knowledge*	*Outcomes: main reasons for using e-cigarettes*		
							*Quit smoking*	*Safety^a^*	*Better experience^b^*
AlBaik et al.^19^ 2014	KSA	3027	All ages	67.7	66.6% of the participants had a Bachelor’s degree or higher	Most participants heard about e-cigarettes; however, less than half thought it was harmful.	✔	✔	-
Karbouji et al.^20^ 2018	KSA	1404	18–60	95	70.8% of the participants were university undergraduates	Awareness of using e-cigarettes was high among participants.	✔	✔	✔
Alfaraj et al.^21^ 2019	KSA	1080	>18	53.8	97.3% of the participants had a high school degree or higher	Most participants were unaware of e-cigarette risks. However, the safety of e-cigarettes was the main concern among participants.	✔	✔	✔
Sabbagh et al.^22^ 2020	KSA	1386	>18	41.2	72.6% of the participants were in high education	There was a lack of knowledge and attitudes regarding e-cigarette use.	✔	✔	-
Abu Khalid et al.^23^ 2021	KSA	865	>18	84.0	NA	A high level of knowledge and awareness regarding the use of e-cigarettes among the participants were reported.	✔	✔	-
Alzahrani et al.^24^ 2021	KSA	465	>18	58.3	70% of the participants had a college degree	There was a lack of awareness among the population related to the effectiveness of e-cigarettes in quitting smoking.	✔	✔	✔
Awan^25^ 2016	KSA	480	Average age 24	63.3	All participants were university students	Many participants aware of e-cigarettes believed that e-cigarettes are less dangerous than traditional cigarettes and could aid smoking cessation.	✔	✔	-
Qanash et al.^26^ 2018	KSA	1007	>15	34.7	All participants were university students	Almost half of the participants believed that e-cigarettes might lead to addiction, and some of them agreed that this method of smoking is harmful.	✔	✔	✔
Almutham et al.^28^ 2019	KSA	256	Average age 22	57.8	All participants were university students	The medical students lack adequate knowledge about e-cigarettes.	✔	✔	-
Alshomrani et al.^29^ 2019	KSA	636	>18	66.7	All participants were medical students	Most e-cigarette usage was not related to quitting among the participants.	✔	✔	✔
Shehata et al.^32^ 2020	KSA	668	18–26	74.9	All participants were university students	Half of the students were knowledgeable regarding e-cigarettes and their effects.	✔	✔	-
Aqeeli et al.^27^ 2020	KSA	775	Average age 21.6	43.3	All participants were university students	Participants were highly aware of e-cigarettes.	✔	✔	✔
Alzalabani and Eltaher^30^ 2020	KSA	527	18–25	59.8	All participants were university students	Participants perceived that e-cigarettes could help people quit smoking and are less harmful and addictive than traditional nicotine smoking.	✔	✔	✔
Habib et al.^31^ 2020	KSA	401	18–23	39	All participants were medical students	Participants’ perception was that e-cigarette use carried a lower risk of serious side effects compared to tobacco smoke.	✔	✔	✔
Natto et al.^33^ 2020	KSA	193	Average age 23	63.8	All the participants were dentistry students	The majority of respondents did not know the potential hazards of e-smoking. In addition, there was low agreement about the addictive effect of e-cigarettes.	✔	✔	✔
Alshanberi et al.^34^ 2021	KSA	910	Mainly <25	55.8	All the participants were medical students	The prevalence of e-cigarette use among medical students was high, and overall knowledge was inadequate.	✔	✔	✔
Alzahrani et al.^35^ 2021	KSA	399	Mean age 21.75±2.91	44.6	All the participants were medical students	There were many misconceptions among participants about addiction and inadequate awareness regarding the harmful effects of e-cigarettes. These opinions have led to unscientific opinions about their use in reduction or in smoking cessation.	✔	✔	✔
Barakat et al.^41^ 2021	Jordan	523	>18	96.4	65.2% of the participants had a Bachelor’s degree or higher	The majority of e-cigarettes users utilize e-cigarettes to help quit smoking.	✔	✔	✔
Abdel-Qader and Al Meslamani^39^ 2021	Jordan	1820	>18	65.9	65.5% of the participants had a Bachelor’s degree or higher	Relatively poor knowledge about the content, regulations, and types of e-cigarettes was reported among participants.	✔	✔	✔
Al-Balas et al.^40^ 2021	Jordan	1536	>18	68.0	NA	About half of the participants believed that e-cigarette use had fewer harmful effects than traditional cigarette smoking. In addition, more than half of the participants believed that e-cigarette use could lead to addiction.	✔	✔	-
Karasneh et al.^38^ 2020	Jordan	400	18–47	95.5	84.2% of the participants had a post-secondary degree	The majority of dual e-cigarette/conventional cigarette users reported stronger perceptions, especially regarding the dangerous health effects of smoking.	✔	✔	✔
Barakat et al.^42^ 2021	Jordan	984	>18	46.8	80.1% of the participants had a Bachelor’s degree or higher	A considerable positive opinion toward e-cigarette use as a helpful aid for tobacco smoking cessation was reported. E-cigarette users perceived this tool to be safer and cheaper alternative to tobacco smoking.	✔	✔	-
Hamadneh et al.^37^ 2021	Jordan	436	Average 31	No males	All the participants were pregnant women	Pregnant smokers believed that e-cigarettes were less hazardous than traditional cigarettes.	✔	✔	-
Al Oweidat et al.^36^ 2020	Jordan	1819	20–25	47	All the participants were medical students	Most of the participants got e-cigarettes. A primary motivation for e-cigarette use appears to be convenient tools to quit or reduce smoking and less harmful than traditional cigarettes.	✔	✔	✔
Khader^45^ 2020	Jordan	320	Average 20.4	17.8	All the participants were university students	The awareness about e-cigarettes among the participants was high, and beliefs about this type of smoking affected their decision to try them.	✔	✔	✔
Alaraj et al.^43^ 2021	Jordan	787	Average 21	75.0	All the participants were university students	Most of the participants had a good knowledge and perceptions regarding e-cigarettes.	✔	✔	✔
Al-Sawalha et al.^44^ 2021	Jordan	1259	>18	29.8	All the participants were university students	The misconception that e-cigarettes help with conventional smoking cessation was the most commonly reported reason for its use among study participants.	✔	✔	-
Abo-Elkheir et al.^50^ 2016	Egypt	1239	15–75	75.4	74.6% of the participants had a secondary degree or higher	More than half of the respondents had heard about e-cigarettes. In addition, more than a third of the participants believed that e-cigarettes help to quit smoking and a third believed that they are less harmful than conventional cigarettes.	NA	✔^‡^	NA
Dwedar et al.^49^ 2019	Egypt	593	18–64	Healthcare providers 25.8	56.0% of the participants had a Bachelor’s degree	Most participants have heard of e-cigarettes. There was high awareness about e-cigarettes among the participants. A more negative attitude about them was among healthcare providers than the general population.	NA	✔^‡^	NA
				General population 44.8	63.9% of the participants had a secondary degree		✔	✔	✔
Kabbash et al.^48^ 2022	Egypt	368	>18	67.4	All participants were university students	The majority of participants had limited knowledge about e-cigarettes.	✔	✔	✔
Barakat et al.^47^ 2021	UAE	392	18–55	70.2	61.0% of the participants had a Bachelor’s degree or higher	Most of the participants believed that e-cigarettes were less harmful than tobacco cigarettes. Many respondents reported shifting towards e-cigarettes as an alternative to conventional smoking.	✔	✔	✔
Ahmed et al.^46^ 2021	UAE	918	>17	29.3	All participants were university students	Reasons for e-cigarette use were to help them quit smoking, enjoy the flavor, and/or believed e-cigarettes use was less harmful than conventional tobacco.	✔	✔	✔
Riahi et al.^52^ 2018	Lebanon^†^	540	>18	48.4	NA	Many participants thought that using e-cigarettes was less harmful than smoking regular cigarettes.	-	✔	-
Aghar et al.^51^ 2020	Lebanon	352	Mean age 30.3±11.8	56.6	72.7% of the participants had a Bachelor’s degree or higher	There was a gap in e-cigarette knowledge, especially among participants who displayed a positive attitude toward e-cigarettes.	✔	-	✔
Palipudi et al.^53^ 2016	Qatar*	8389	>15	NA	NA	Around half of the participants had a high level of awareness related to e-cigarette use.	✔	-	-
Kurdi et al.^54^ 2021	Qatar	199	Mean age 23.4±6.8	37.2	All participants were university students	The participants had knowledge gaps and misconceptions about the harms of e-cigarette use, especially among the users.	✔	✔	✔

a Safer than tobacco cigarettes and other types of smoking methods related to health and causes of health problems.

b Compared mainly to conventional tobacco smoking.

‡ These studies were conducted among people who did not use e-cigarettes.

† This study was performed in 13 countries. The results included in the table are related to Lebanon only.

* This study was performed in four countries. The results included in the table are related to Qatar only. NA: not available. KSA: Kingdom of Saudi Arabia. UAE: United Arab Emirates.

## PREVALENCE AND AWARENESS OF E-CIGARETTES IN ARAB COUNTRIES

### Saudi Arabia

Most of the studies (n=17) included in this review were conducted in Saudi Arabia^19-35,55^. The first study was conducted in 2014 (participants, n=3027). A small number of participants (4.1%) had used e-cigarettes daily; however, less than half of the participants reported that these could be harmful. Less than 20% of the participants reported that the main reason for using e-cigarettes was to quit smoking^19^. In five other studies conducted among the general population, between 31.8% to 77.6% of the participants were e-cigarette users^20-24^. In addition, the participants were either only e-cigarette or dual users (using e-cigarettes with another smoking product). Generally, the main reason for using e-cigarettes was to quit smoking, as the participants claimed that e-cigarettes could be safer than traditional tobacco smoking. Interestingly, there were also concerns regarding the safety of e-cigarettes among the participants in three studies.

In Saudi Arabia, the use of e-cigarettes among the general population was 2.2% and among the smoking population was 9.6%, which gave it the third smoking method in the country after cigarettes and hookah^55^.

Eleven studies were conducted among university students in Saudi Arabia, where the average age of the participants was <26 years. The findings of these studies indicated that most participants had heard about e-cigarettes, and 5.7–51.4% of them were e-cigarette users. In addition, between 31.0 and 42.7% of the participants reported that they used e-cigarettes to help in quitting smoking. Although the participants in these studies had reported that e-cigarettes were harmful, they also reported that e-cigarettes were ‘less dangerous’ than traditional tobacco smoking. Generally, male students were more likely to use e-cigarettes than females, including medical students^25-35^.

### Jordan

E-cigarettes use was legalised officially in Jordan in 2019^56^. There were ten published studies related to e-cigarettes^36-45^. Three studies were conducted among the general population in Jordan. The participants were found to be familiar with e-cigarettes and thought they could use e-cigarettes as an alternative to traditional smoking^39-41^. The prevalence of e-cigarette use ranged between 11.7% and 39.2% in these three studies. In addition, the prevalence of e-cigarette use was more among males than females. Relatively poor knowledge of the content, regulations and types of e-cigarettes was reported among the participants. The general opinions of the participants were that e-cigarettes might have the same or some harmful effects as traditional cigarettes and e-cigarettes could lead to nicotine addiction in the future.

Four studies were conducted among university students^36,43-45^. The first study was conducted in 2017, and the use of e-cigarettes was low (2.2%) among students^45^. However, the use of e-cigarettes rose by almost eight- to ten-fold, respectively, in studies published 3 to 4 years later^36,43,44^. Generally, the primary motivations to use e-cigarettes were the beliefs that e-cigarettes do not cause dependence, are not as harmful as tobacco smoking, as well as the perception that e-cigarettes could help with quitting smoking. In addition, dual-use was common in these studies. In the two other studies among current and former e-cigarette users, the dual users were 56.7% in the first study and 17.8% in the second study. More than half of the participants believed that e-cigarettes could reduce the cravings for smoking tobacco and help with smoking cessation^38,42^. Another reason to use e-cigarettes was that most participants believed e-cigarettes are more socially acceptable than smoking traditional cigarettes, encouraging smokers to continue smoking but possibly using a low nicotine dose^43^.

In the Hamadneh et al.^37^ 2021 study conducted among pregnant women (n=436), smoking was high (10.3% for e-cigarettes alone and 55.8% for dual users). The level of awareness regarding the long-term consequences of tobacco use was found to be low among the participants. Non-smoking pregnant women believed that e-cigarettes were as bad for their health as cigarettes, while smoking pregnant women believed that e-cigarettes were less hazardous than cigarettes.

Finally, only one study assessed beliefs toward smoking and COVID-19 infection (n=2424), which included e-cigarette users (9.4%) and 12.9% were dual users. More than a third of respondents (38.2%) believed that e-cigarette use is associated with a risk of contracting COVID-19 infection. A low percentage of participants (21.6%) indicated that e-cigarettes are a safer alternative to other forms of tobacco products during the COVID-19 pandemic^57^.

### UAE

The use of e-cigarettes was legalised in the UAE in April 2019^58^. The prevalence of e-cigarette use among the general population in the UAE was 3.8% in 2020^59^. The prevalence was higher (8.8%) among university students, including dual users, and males were more common users of e-cigarettes than females^46^. In another study conducted among current and former e-cigarette users, more than a quarter of participants were dual users^47^. The main reasons for using e-cigarettes were the belief in their safety and enjoyment due to the availability of a wide range of e-liquid flavors^46,47^.

### Egypt

Although e-cigarettes are not officially legalised in the Egyptian market, they can be purchased in Egypt. In a study conducted between 2015 and 2017 among young adults who had a history of waterpipe use in Egypt (n=2014), e-cigarettes were used by 10.4% of the participants. In addition, young female waterpipe smokers who also used electronic nicotine delivery systems (ENDS) were more than ten times the young males (51.2% vs 4.1%)^60^. Another two studies conducted among university students reported that e-cigarette prevalence was between 10.6 and 16.5%^48,61^. At the same time, more than half of those who vaped also used other smoking products^61^.

Studies related to knowledge and beliefs about e-cigarettes among non-users of e-cigarettes were also conducted in Egypt. In a study among healthcare providers and the general population, the participants believed that e-cigarettes were not safe, not an effective smoking cessation strategy, and can encourage smoking habits. They also believed that e-cigarettes contained chemicals that may cause health issues in the long-term. However, the general population arm of the study showed low awareness of the potential harms of e-cigarettes among participants^49^. A study by Abo-Elkheir and Sobh^50^, which included the general population, revealed a high level of awareness and positive perception among participants about using e-cigarettes as a smoking cessation device.

In a university study (n=368), the prevalence of vaping was 10.6%. Moderate to high knowledge regarding e-cigarettes with male predominance was shown, and the most frequent reason was keeping with fashion followed by the influence of peers. Only 4.90% of smokers ever tried to quit smoking e-cigarettes^48^.

### Kuwait

The use of e-cigarettes was reported in two studies among the adolescent population aged 16–18 years (with n=1525 and n=1345, respectively) in Kuwait^62,63^. In these two studies, more than a quarter of the participants were found to be e-cigarette users. In addition, a high percentage of the smokers (84.8%) were dual users, and male adolescents were more likely to use e-cigarettes than females^62^. In Kuwait, the sales of e-cigarettes are banned^64^.

### Lebanon

In 2015, regulations for e-cigarette imports were put in place in Lebanon by the Ministry of Finance^65^. A study by Aghar et al.^51^ reported that the use of the e-cigarettes among participants (n=352) was 11.5%, and most participants (>80%) believed that using e-cigarettes is less harmful or not harmful compared with traditional cigarettes. In addition, more than half of the participants also thought that e-cigarettes could help people cut down on cigarettes or quit smoking^51^. In another study, most participants believed that e-cigarettes were less harmful than traditional cigarettes^52^, and in another study, 8% of the participants reported current use of e-cigarettes^66^.

### Qatar

Although the personal use of e-cigarettes is not prohibited in Qatar, the sale, distribution and/or possession of e-cigarettes in large quantities are illegal^67^.

In two studies about e-cigarette use among the general population in Qatar, the prevalence of e-cigarette users was almost similar at 1.6% and 2.0%, and the majority of those who vaped (>82%) were dual users^53,68^. In the third study, among university students, the prevalence was higher (14.0% of the participants)^54^. Furthermore, the overall prevalence of using e-cigarettes was higher among males than females. As per the published articles in Qatar, e-cigarette users perceived e-cigarettes to be less harmful than other tobacco smoking. In addition, e-cigarette users believed that e-cigarettes could help reduce smoking, and quitting smoking was a reason to use e-cigarettes by the participants^53,54^. Moreover, the availability of various flavors and the lack of restrictions on using e-cigarettes in public places were reported in a study as factors that may increase this type of smoking among the population^54^.

### Tunisia

There were two published conference abstracts on the use of e-cigarettes in Tunisia. In the first abstract in 2020, among healthcare workers (n=414), 4.8% of the participants were e-cigarette users. Their reasons to use e-cigarettes were for pleasure and to reduce smoking or quit smoking^69^. Furthermore, a study by Maalej et al.^70^ demonstrated that 20.5% of high school students (aged 15–20 years) were e-cigarette users. They mentioned that the main reason for using e-cigarettes was feeling more sociable. They were more confident in using such a modern smoking method and perceived less harmful effects than traditional smoking products by lowering the nicotine doses^70^.

### Sales and consumptions regulations of e-cigarettes in the Arab countries

Various regulatory approaches to the use of e-cigarettes have been implemented in Arab countries. Several countries, such as Bahrain, Lebanon, Oman, and Qatar, have banned the sale of e-cigarettes (Table 2)^50,67,71-74^. However, in some of these Arab countries, it is legal to consume e-cigarettes, but it is illegal to sell e-cigarettes^75^. In addition, there are many shops, such as in Bahrain and Kuwait, that sell e-cigarettes to people, as noted when searching through Google (Online shops).

**Table 2:** E-cigarette regulation in Arab countries

*Regulation*	*Country*
Banned	Bahrain, Kuwait, Lebanon, Oman, and Qatar
Legal use (year)	Jordan (2019)^37^, Saudi Arabia (2019)^76^, and UAE (2019)^48^
Unknown	Algeria, Egypt, Iraq, Libya, Mauritania, Morocco, Palestine, Sudan, Syria, Tunisia, and Yemen

Only three countries, Jordan, Saudi Arabia and the UAE, have passed regulations to control the sales and use of e-cigarettes^56,57,76^. These regulations controlled the use of e-cigarettes similar to traditional tobacco products by applying taxes on products, standard manufacturing procedures, smoke-/vape-free areas, and restricting the sales of e-cigarettes to adults aged >18 years.

Some countries such as Bahrain, UAE, Kuwait, and Saudi Arabia have introduced a 100% excise tax on e-cigarettes, while Lebanon and Jordan have introduced a 150% excise tax^65,77-81^. In addition, there is a tax on e-cigarette products in some countries, such as Egypt (44% of its value) and Morocco^82,83^.

In other Arab countries, although there are regulations regarding the trade and sale of tobacco products (including traditional cigarettes, tobacco for hookah, etc.), to our knowledge, there are no specific regulations relating to e-cigarette sales. There are also internet websites for selling e-cigarettes in some of these countries. In other countries, regulations regarding smoking treat users of e-cigarettes in the same way as smokers of traditional cigarettes. They banned the sale of e-cigarettes to minors and/or restricted public use of e-cigarettes^84^. Some of these smoking regulations specify that e-cigarette tools are under the same rule, or the regulations are applied to all types of smoking products with no particular policy for e-cigarettes^85-89^. In addition, all published smoking regulations in Arab countries ban advertising any nicotine products, including e-cigarettes^56,57,76,84,85^.

## DISCUSSION

The prevalence of using e-cigarettes among people in Arab countries varied; however, the prevalence of e-cigarette use has been steadily increasing in the past few years, especially among young males. In addition, e-cigarette use was reported in most studies among females and people aged <18 years. There were similarities between sociodemographic factors (such as gender and age) of e-cigarette users and reasons for smoking e-cigarettes among people in Arab countries. Most participants in several studies were educated with graduate and postgraduate degrees^19,20,88^.

The reasons for vaping and awareness of e-cigarettes vary between countries. The main reasons for using e-cigarettes were the willingness to quit smoking, the perception that e-cigarettes could be safer alternatives to traditional cigarettes, and the use of more modern smoking products. In addition, some studies reported that people believed e-cigarettes have risks but less than other types of smoking^54,89^. Furthermore, awareness and knowledge among the Arab populations (the general population and university students) in most of the studies were high regarding the risk of e-cigarettes on the health of cigarette users. High levels of awareness were expected as the participants in these studies mostly held undergraduate and postgraduate degrees. In addition, most of the participants were young. In addition, most studies reported that social media are the main source of information related to e-cigarette use^21,24,27,28,32,39,41,43,47,51,88^. Furthermore, most of the studies used electronic questionnaires, thus, respondents would be participants who have access to the Internet. This high awareness was also found among healthcare providers, for whom this issue is part of their daily work, in addition to their having access to reliable sources of information.

The findings of these studies related to the use of e-cigarettes in Arab countries are in line with other published review articles among populations in other regions of the world, such as China, European countries, and the United States^6,90,91^, as they identified the association between sociodemographic factors and the use of e-cigarettes. For example, there was increased use of e-cigarettes among adolescents and young adults and it was reported to be higher among males than females. In addition, the prevalence of e-cigarette use has been reported to be highly prevalent among college students.

In several Arab countries, the prevalence of e-cigarettes was 5% or higher among the general population and university students who participated in the described studies^20,25-28,30-33,39-41,46-48,51,61,70,88^. The prevalence was lower in European countries (ranging from 1% to 2.9%), China (0.5 to 3.1%) and the USA (2.8% in 2017 among adult groups, and 5.2% among the young adult population in 2015)^6,90,91^. This may be explained by the methodology of the included studies, which are cross-sectional, in the current review that involved educated people and university students. Furthermore, the level of awareness regarding the relationship between the desire to quit smoking and e-cigarettes among participants has been highlighted in most studies.

### Reasons for using e-cigarettes among Arab communities

Overall, e-cigarettes are becoming popular among Arab communities. Prevalence of using e-cigarettes, for instance, was particularly high (>25%) among smokers in countries such as Jordan, Lebanon, Egypt and Saudi Arabia^20,24,41,51,60^. In the Arab countries, the main reason for using e-cigarette devices among participants in most studies (Table 1) was positive perceptions of e-cigarette safety with minimal harmful health effects. So, e-cigarettes were considered a safer alternative compared with other methods of tobacco smoking, despite the lack of evidence on the long-term effects of e-cigarettes.

Although the long-term abstinence versus relapse among those who quit smoking with the help of e-cigarettes is debated, the desire to quit smoking among the participants (in 20 studies) was one of the main reasons for using e-cigarettes. Another reason was related to the characteristics e-liquids of e-cigarettes, such as flavors and smell, including the fact that e-cigarettes do not leave an unpleasant odor like traditional cigarettes, and nicotine content. Non-smoker participants also thought that e-cigarettes were less harmful than smoking and could help to quit smoking.

Most of the studies in this review reported that the social media were one of the main sources of information regarding e-cigarettes among the participants^21,24,27,28,32,39,41,43,47,51,88^. This was reported in many studies worldwide^91^. This is mainly due to the high level of social media use among adolescents and young adults, mainly related to the increased promotion of e-cigarette use. In addition, e-cigarette use among friends was a significant factor in using this device, as many participants reported peer influence in their initiation of e-cigarette use^24,25,27,28,30,32,35,39,43,65,87^. The peer influence was significantly associated with an increased probability of e-cigarette use among adolescents^6,91^. The cost of e-cigarettes was not assessed in most of the studies. However, three studies stated that the lower cost of e-cigarettes compared with other cigarette types might be a motivation factor for e-cigarette use^27,30,51^.

### Age and gender

The prevalence of e-cigarette use was high in several studies among participants aged <25 years^21,24,25,27-33,38,45,48,61^. In many of these studies, e-cigarette use among young adults (<25 or <30 years) was higher than among older people^19,20-22,39,41,50,60^. There are similarities in these findings according to the data reported in several published reviews^6,90,91^.

In all Arab countries, males smoke traditional cigarettes more than females^2,17^. The included studies in the current review indicate that e-cigarette use was more prominent in males than females^19,21,24,26,30,31,36,39,47,51,53,55,62^. Only one study, conducted in Qatar, showed no difference between males and females in e-cigarette use^54^. Generally, knowledge about e-cigarettes was found to be higher among males than females in several studies. This knowledge and awareness related to less harm than traditional cigarettes and as a smoking cessation aid. The differences in knowledge and awareness were mainly related to more adult males using this form of smoking in the studies. Most of them were educated at college level or higher. In addition, males, to a greater extent, use e-cigarettes as a tool for helping them to quit smoking tobacco cigarettes.

### Dual users

E-cigarettes have been marketed as healthy alternatives to traditional cigarettes or as a smoking cessation aid. However, most e-cigarette users do not stop smoking and instead use both e-cigarettes and traditional cigarettes or hookah (dual users). Dual users were reported in many of the included studies in this review^20-22,24,26,36-38,41,46-48,51,53,60,61,68^. The use of e-cigarettes with other types of tobacco was more common than the use of e-cigarettes alone. In many cases, smokers might switch to e-cigarettes and quit traditional cigarettes, but they might fail in that and become dual users^92,93^.

Recent studies have shown that dual users are associated with an increased risk of cardiovascular diseases compared to cigarette smokers^94^. However, participants in several studies reported that they tried to quit using e-cigarettes and considered e-cigarettes safer than conventional cigarettes. This is mainly because most people have low smoking experience and have a low level of knowledge regarding the dangers of nicotine smoking and smoking cessation plans.

### Limitations

The findings are limited by the quality of studies and methods/surveys, mainly cross-sectional electronic surveys, used in the included studies. Therefore, the data extracted from these studies were limited to the time of studies. The electronic distribution of surveys in several studies likely limits the generalizability of the findings as the main individuals who have access to the internet would respond to the survey. In addition, many studies included in the review did not provide information about the psychometric properties of the instruments used in the surveys. Thus, they did not capture the participants’ true prevalence and awareness of e-cigarette use.

A lack of a unified definition of variables such as the economic status of participants, smoking status, including the daily use volume of e-liquid, and nicotine content in using these devices and presenting other variables, made it difficult to find more information related to e-cigarette users. Furthermore, the wording of questions assessing e-cigarette use may differ in several studies, potentially introducing misclassification bias. For example, in some populations, the term ‘vape’ is more popular than e-cigarettes.

## CONCLUSION

The popularity of e-cigarettes in several Arab countries has increased in the last decade, specifically in the last couple of years and particularly among younger adults. Handheld e-cigarette devices are gaining wide acceptance as they are marketed to be easy to use with a range of flavours and less harmful than traditional smoking. In addition, there is a lack of a clear policy to regulate sales and consumption in many the Arab countries. Finally, age, gender, education level, and cigarette smoking (dual) were associated with awareness of e-cigarettes. Accordingly, there is a strong need to implement tighter control rules on the trade and sales of e-cigarettes until more evidence becomes available. In addition, there is a need for educational programs that should emphasise the potentially harmful and addictive properties of e-cigarettes.

## Data Availability

The data supporting this research are available from the corresponding author on reasonable request.
